# A systematic scoping review protocol to summarise and appraise the use of artificial intelligence in the analysis of digital videos of invasive general surgical procedures

**DOI:** 10.1097/SP9.0000000000000012

**Published:** 2023-10-30

**Authors:** Anni King, George Fowler, Rhiannon C. Macefield, Fang-Fang Quek, Hamish Walker, Charlie Thomas, Sheraz Markar, Jane M. Blazeby, Natalie S. Blencowe

**Affiliations:** a National Institute for Health Research Bristol Biomedical Research Centre (Surgical Innovation Theme), Centre for Surgical Research, Bristol Medical School: Population Health Sciences, University of Bristol; b Department of Surgery, North Bristol NHS Trust, Bristol; c Nuffield Department of Surgery, Oxford University Hospitals NHS Foundation Trust, Oxford, Oxfordshire, UK

**Keywords:** artificial intelligence, operative, procedures, review, surgery, video

## Abstract

**Background:**

Intraoperative video recordings are a valuable addition to operative written documentation. However, the review of these videos often requires surgical expertise and takes considerable time. While a large amount of work has been undertaken to understand the role of artificial intelligence (AI) in healthcare more generally, the application of these techniques to automate the analysis of surgical videos is currently unclear. In this systematic scoping review, we sought to give a contemporary overview of the use of AI research in the analysis of digital videos of invasive general surgical procedures. We will describe and summarise the study characteristics, purpose of the applications and stage of development, to ascertain how these techniques might be applied in future research and to identify gaps in current knowledge (e.g. uncertainties about the study methods).

**Methods:**

Systematic searches will be conducted in OVID Medline and Embase, using terms related to ‘artificial intelligence’, ‘surgery’ and ‘video’ to identify all potentially relevant studies published since 1st January 2012. All primary studies where AI has been applied to the analysis of videos (recorded by conventional digital cameras or laparoscopic or robotic-assisted technology) of general surgical procedures will be included. Data extraction will include study characteristics, governance, details of video datasets and AI models, measures of accuracy, validation and any reported limitations.

**Ethics and dissemination:**

No ethical approval is required as primary data will not be collected. The results will be disseminated at relevant conferences, on social media and published in a peer-reviewed journal.

## Introduction

Highlights

- This work will generate an in-depth understanding of the current reported uses of artificial intelligence (AI) to assist with the automated analysis of intraoperative videos.
- Rigorous and comprehensive search methods will be applied to identify a wide range of diverse data sources for inclusion in the review.
- A summary of existing relevant literature will provide a valuable aid to those looking to undertake future research into this field.
- The review will identify current limitations in AI analysis methods.

### Background

Intraoperative videos are a rich source of data, providing a comprehensive and unbiased insight into surgical practice. They complement the information contained within operative notes, documenting what actually happened as well as what was reported or recalled^1–3^. Surgical videos have the potential to facilitate a greater understanding of the surgical workflow via, for example, identification of key surgical steps and performance metrics such as procedural delays, and adverse or unexpected events^4^. Technological advances (for example, laparoscopic and robotic-assisted techniques) have increased exponentially, allowing for more convenient surgical filming and dissemination of intraoperative data^5^. Such innovations have provided surgeons with compatible equipment to film procedures with relative ease of access and data transfer^6^. Despite this, however, the process of manually reviewing and assessing surgical video recordings requires experienced clinicians and is time and resource intensive. To overcome these issues, there is increasing interest in the application of artificial intelligence (AI) to facilitate automated video analysis.

The term AI refers to the concept of machines or computers demonstrating behaviour and performing tasks commonly associated with human intelligence^7^. AI has the capability to facilitate the automated analysis of a diverse range of textual, imaging and audio data^8^. AI has been applied widely throughout healthcare, from interpretation of diagnostic imaging^9^, classification of cancer^10^, to interactive chatbots delivering mental health support^11^. Automating the extraction and/or analysis of information from surgical videos has the potential to benefit clinical processes and improve safety and efficiency via enhanced intraoperative visualisation^12^ and real-time detection of adverse events^13^. Early-stage clinical AI evaluation will play an important role in evaluating the clinical utility, safety and human factor challenges in a real clinical setting (DECIDE-AI) and reporting guidelines now exist to facilitate the appraisal of these studies^14^.

## Aims and objectives

To the authors’ knowledge, there has been no systematic scoping review examining the use of AI to facilitate automated analysis of surgical videos. This study therefore aims to summarise the use of AI in the analysis of operative videos, and identify evidence gaps. Specifically, we sought to describe the study characteristics, with attention to the datasets (number of videos, surgeons and centres contributing to the video dataset), purpose of the applications and stage of development. The findings of this review will help inform future research, identifying potential areas of automated video analysis and evidence gaps, including the source and size of datasets, annotation software used and the level of expertise to annotate the videos.

## Methods

This systematic scoping review was designed in accordance with the Preferred Reporting Items for Systematic Reviews and Meta-analysis extension for Scoping Reviews (PRISMA-ScR).

### Identifying relevant records

A comprehensive electronic search of OVID Medline and EMBASE will identify all potentially relevant studies published since 1st January 2012. This cut-off (which has previously been adopted)^15^ was applied to account for advancements in machine learning, with the development of deep learning^16^. Searches will be developed in collaboration with an expert subject librarian, using free text and Medical Subject Headings related to ‘artificial intelligence’, ‘surgery’ and ‘video’. Database search results will be imported into electronic software^17^ to facilitate identification and removal of duplicate records and the management of the reviewer screening.

#### Inclusion and exclusion criteria

Primary research studies, where AI has been applied to facilitate the analysis of intraoperative videos (conventional digital, robotic-assisted or laparoscopic recordings) of invasive general surgical procedures, will be included. For the purposes of this review, a video will be defined as a programme, movie or other visual media featuring moving images, with or without audio https://www.dictionary.com/browse/video. An invasive surgical procedure will be defined as a procedure where purposeful/deliberate access to the body is gained via an incision^18^. Intraoperative videos recorded with devices inserted into natural orifices (e.g. endoscopy, cystoscopy) will be excluded, as they do not meet the definition of an invasive surgical procedure and several reviews have already appraised the role of AI in this field^2–6^. Studies involving AI applied to types of imaging other than conventional digital video, robotic-assisted and laparoscopic videos will not be included (e.g. videos recorded via surgical microscopes, X-ray and ultrasound imaging, etc.).

Secondary research (i.e. systematic reviews), editorials, conference abstracts and papers, and non-English publications will be excluded. Studies will be limited to those carried out in humans. For the purpose of this review, studies exploring the use of augmented reality will also be excluded, to maintain a manageable amount of papers.

### Study selection

Assessment of study eligibility will be a two-stage process. First, abstracts identified from the initial database search will be screened for eligibility by at least two independent reviewers. Full texts of potentially relevant articles will subsequently be reviewed by at least two independent reviewers. Conflicts will be resolved through team discussions. Reference lists of all included studies will be examined to identify any additional relevant articles that may have been missed.

### Data extraction

Included studies will undergo data extraction using a predesigned electronic form. Data relevant to the following categories will be extracted:

1. *Study characteristics*: author, journal, year of publication, country of origin, surgical specialty, operation(s) of interest, and the overall objective of AI assessments.
2. *Governance:* Reporting of ethical approval, regulatory approval (e.g. Medicines and Healthcare products Regulatory Agency), funding and patient consent to imaging.
3. *Characteristics of dataset*: data source (i.e. publically available video dataset, pre-existing private collection, or videos collected during the study), number of patients, number of videos, and number of surgeons/centres contributing to video dataset.
4. *Input features*: modality of video (e.g. laparoscopic, robotic-assisted or conventional digital video), nature of the AI model(s) applied, size of training/test/validation datasets (if relevant), method of AI model training, whether a comparator group was used, and whether any assessments were undertaken in real-time.
5. *Outcomes*: measures of accuracy [e.g. sensitivity, specificity, positive predictive values and area under the receiver operating characteristic curve (AUC)] and other recorded outcome measures (e.g. time).
6. *Stage of development:* stage of evaluation as defined by the IDEAL framework^19^.

### Data synthesis

Study characteristics will be descriptively summarised and presented in tabulated format. Data relating to study characteristics (e.g. publications, video datasets and objective of AI model) will be grouped into categories and presented in a narrative summary. The findings will be reported by specific AI objective(s), which may include, but are not limited to: surgical phase recognition, instrument recognition, skill or task analysis, anatomy classification and enhanced intraoperative visualisation. While a few AI-specific assessment tools exist, and several more are in development, none are appropriate for this systematic scoping review^20,21^. Therefore, we do not plan to perform a quality assessment or risk of bias assessment.

## Ethics and dissemination

No ethical approval is required as primary data will not be collected. The results will be disseminated at relevant conferences, on social media and published in a peer-reviewed journal.

## Limitations

This study will not address studies written in languages other than English, resulting in literature in non-English studies being unaddressed in this review.

## Ethical approval

Not applicable.

## Consent

Not applicable.

## Funding information

This study was supported by the National Institute for Health and Care Research (NIHR) Biomedical Research Centre (BRC) at the University of Bristol (BRC-1215-20011). The views expressed in this publication are those of the authors and not necessarily those of the NHS, the NIHR, the Royal College of Surgeons of England or the Department of Health and Social Care. NSB is funded by an MRC Clinician Scientist Fellowship (MR/S001751/1).

## Author contribution

A.K.: conceptualisation, project admin, data collection, writing – original draft, review and editing; G.F.: conceptualisation, data collection, writing – original draft, review and editing; N.S.B.: conceptualisation, data collection, writing – original draft, review and editing, and funding acquisition; R.C.M.: data collection, original draft, review and editing; F.-F.Q., H.W. and C.T.: data collection; S.M.: review and editing; J.M.B.: conceptualisation, review and editing.

## Conflicts of interest disclosure

There are no conflicts of interest.

## Research registration unique identifying number (UIN)

Not applicable.

## Guarantor

Natalie Blencowe.

## Data availability statement

Not applicable.

## Provenance and peer review

Not invited.
